# [Retracted] Puerarin inhibits apoptosis and inflammation in myocardial cells via PPARα expression in rats with chronic heart failure

**DOI:** 10.3892/etm.2025.12916

**Published:** 2025-06-30

**Authors:** Le He, Tong Wang, Bing-Wei Chen, Feng-Min Lu, Jing Xu

Exp Ther Med 18:3347–3356, 2019; DOI: 10.3892/etm.2019.7984

Following the publication of this paper, it was drawn to the Editor’s attention by a concerned reader that all the histological images shown in Fig. 5 on p. 3352 were strikingly similar to images that had already appeared in Figs. 10 and 11 in a paper written by different authors at different research institutes in the journal *Drug Design, Development and Therapy.* Upon performing an independent analysis of the data in the Editorial Office, it came to light that some of the data shown for the TUNEL assay experiments in Fig. 3A had similarly already appeared in an article written by different authors in the journal *BMC Cardiovascular Disorders*.

In view of the fact that the abovementioned data had already apparently been published previously, the Editor of *Experimental and Therapeutic Medicine* has decided that this paper should be retracted from the Journal. After contacting the authors, they accepted the decision to retract the paper. The Editor apologizes to the readership for any inconvenience caused.

